# Circular METRN RNA hsa_circ_0037251 Promotes Glioma Progression by Sponging miR-1229-3p and Regulating mTOR Expression

**DOI:** 10.1038/s41598-019-56417-8

**Published:** 2019-12-24

**Authors:** Qinchen Cao, Yonggang Shi, Xinxin Wang, Jing Yang, Yin Mi, Guan Zhai, Mingzhi Zhang

**Affiliations:** 1 Department of Radiation Therapy, The First Affiliated Hospital of Zhengzhou University, Zhengzhou, 450052 People’s Republic of China; 2 Department of Neurology, The First Affiliated Hospital of Zhengzhou University, Zhengzhou, 450052 People’s Republic of China; 3 Department of Neurosurgery, The First Affiliated Hospital of Zhengzhou University, Zhengzhou, 450052 People’s Republic of China; 4 Department of Oncology, The First Affiliated Hospital of Zhengzhou University, Zhengzhou, 450052 People’s Republic of China

**Keywords:** CNS cancer, CNS cancer

## Abstract

Circular RNAs (circRNAs) are a newly identifed non-coding RNA in many cellular processes and tumours. This study aimed to investigate the role of *hsa_circ_0037251*, one circRNA generated from several exons of the gene termed *METRN*, in glioma progression. Through *in vitro* experiments, we discovered that high expression of hsa_circ_0037251 was related to low expression of the microRNA *miR-1229-3p* and high expression of mTOR. The over-expressed hsa_circ_0037251 promoted cell proliferation, invasion and migration in glioma, while knockdown of hsa_circ_00037251 promoted cell apoptosis and induced G1 phase arrest. Then, hsa_circ_0037251 was observed to directly sponge miR-1229-3p, and mTOR was identified as a direct target of miR-1229-3p. In addition, knockdown of hsa_circ_0037251 up-regulated the expression of miR-1229-3p and inhibited the expression of mTOR. And overexpression of miR-1229-3p or low-expressed mTOR inhibited the glioma cell progression. Furthermore, transfection with mTOR overexpression vectors can restore the abilities of glioma cell progression even if hsa_circ_00037251 was knocked down using siRNAs. *In vivo* experiments revealed that hsa_circ_00037251 promoted the growth of xenografted tumours and shortened the survival period. These results indicated that hsa_circ_0037251 may act as a tumour promoter by a hsa_circ_0037251/miR-1229-3p/mTOR axis, and these potential biomarkers may be therapeutic targets for glioma.

## Introduction

As the most common primary malignant tumour in the central nervous system, glioma accounts for 27% of all brain tumours and 80% of all malignant brain tumours^1–3^. Glioblastomas represent more than 50.0% of all malignant gliomas and have increased morbidity and high mortality rates^3^. Despite significant advances in elucidating the biological mechanisms of these tumours and even radical surgical resection followed by adjuvant radiotherapy and/or chemotherapy, patients have a poor clinical prognosis with a median survival time of less than 15 months following diagnosis and aggressive treatment^2^. In addition, the exact cause of glioma is still unclear^1,4^. Given these findings, therapeutic approaches with novel targets must be identified to prevent these malignancies and to improve patient survival.

Circular RNAs (circRNAs) were first observed by Hsu and Coca-Prados in eukaryotic cells using electron microscopy^5^ and subsequently were found in yeast mitochondria^6^. Owing to their conservative sequences, circRNAs may have potential roles in regulating of pathogenesis^7^, including carcinogenesis and tumour development^8–15^. Concretely, circRNAs are involved in the occurrence of many cancers such as hepatocellular carcinoma, gastric cancer, colorectal cancer and so on^16^. Moreover, circRNAs have been verified as “microRNA (miRNA) sponges”^11–13^, harboring multiple miRNAs and functioning as miRNA inhibitors^14,15^. These circRNA-miRNA regulatory networks affect target genes, which ultimately regulate cancer development and metastasis. Current studies have also revealed that some circRNAs promote glioma carcinogenesis by decreasing the expression of miRNAs^11,12^. However, the exact mechanisms of the circRNA-miRNA network on target genes involved in cell proliferation, apoptosis, cell cycle regulation, invasion and migration remain unknown in glioma. More intensive studies on glioma progression are necessary.

Hsa_circ_0037251 is a circRNA generated from several exons of the gene encoding the meteorin protein (METRN), which was reported to play roles in both proliferation^17^ and differentiation^18^ of glioblasts. In preliminary experiments, we generated deep RNA sequencing data from glioma samples and their paired adjacent normal tissues and identified miRNA and gene candidates, and we found that hsa_circ_0037251 was over-expressed in glioma tissues. In contrast, miR-1229-3p was expressed at low levels in glioma tissues. The relationship between hsa_circ_0037251 and miR-1229-3p in glioma progression requires further investigation. We also focused on the most abundantly and differentially expressed genes. For instance, mammalian target of rapamycin (mTOR) protein is a highly conserved serine/threonine kinase that belongs to the PI3K-related kinase family^19^. mTOR pathways were shown to be up-regulated in cell proliferation in brain tumours^20^. The downregulation of miRNAs was associated with glioblastoma cell malignancy via the mTOR signaling pathway^21^. Coincidently, by bioinformatic analysis, we found that both hsa_circ_0037251 and the 3′-untranslated region (UTR) of mTOR share miRNA response elements (MREs) of miR-1229-3p, which suggested an association between hsa_circ_0037251 and mTOR in glioma.

Therefore, we hypothesized that hsa_circ_0037251 might be involved in glioma progression by influencing the expression of mTOR in a miRNA-mediated manner. Biological and molecular experiments *in vitro* or *in vivo* were conducted to address this speculation.

## Results

### The over-expressed hsa_circ_0037251 promoted cell proliferation, invasion and migration in glioma, while knockdown of hsa_circ_00037251 promoted glioma cell apoptosis, induced G1 phase arrest

**Figure 1:**
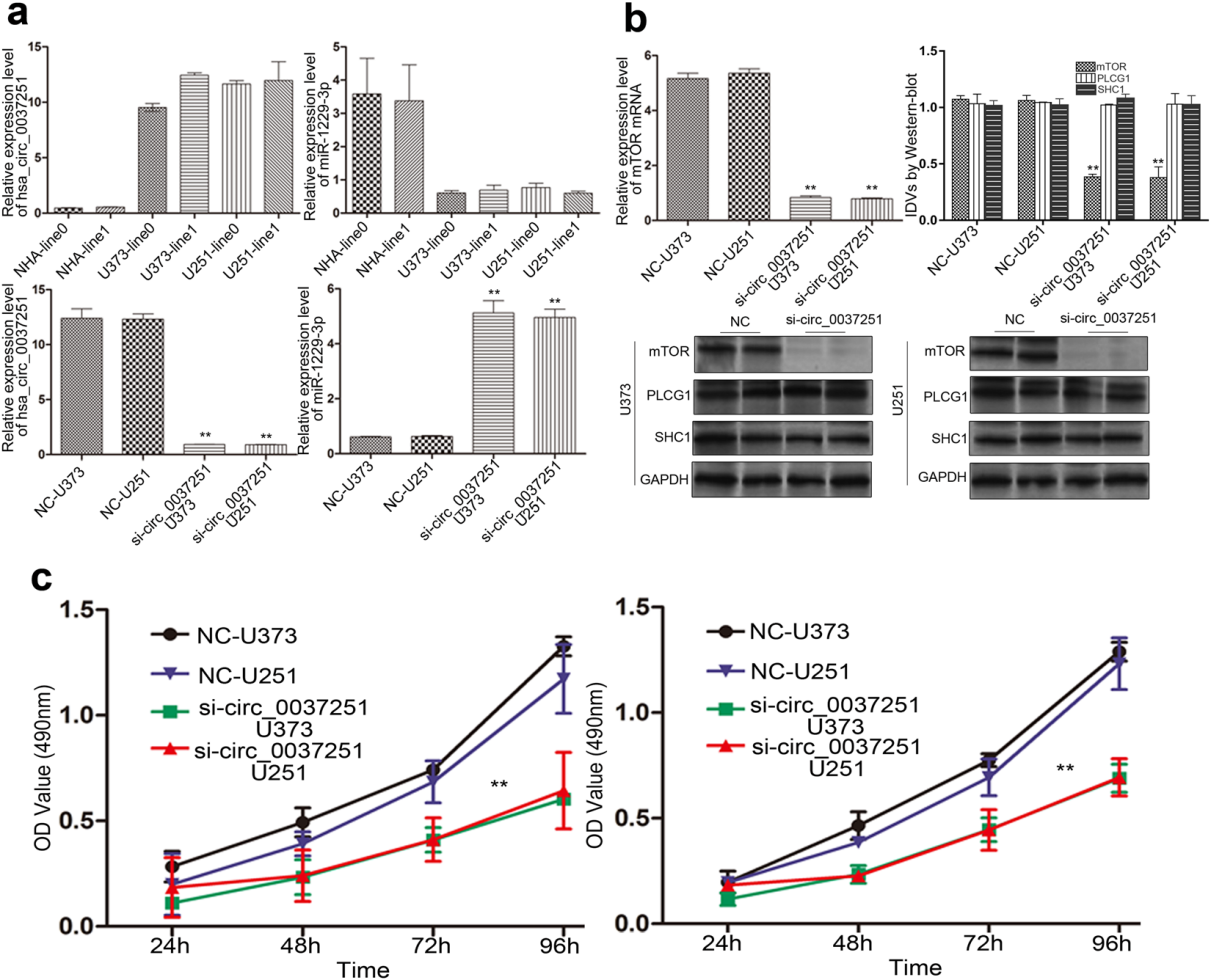
Hsa_circ_0037251 was over-expressed in glioma cell lines, and the over-expressed hsa_circ_0037251 was related to the promoted cell proliferation while knockdown of hsa_circ_00037251 inhibited cell proliferation. (**a**) The expression of hsa_circ_0037251 was significantly up-regulated in glioma cell lines compared normal human astrocytes (NHA) cell lines. *Line0* represents the cell line that has just been obtained but has not been subcultured in our laboratory, and *line1* represents the cell line that has been stably subcultured. There were 6 technical replicates in each biologically replicated group (*line0* or *line1*), respectively. (**b**) Knockdown of hsa_circ_00037251 promoted the expression of miR-1229-3p and inhibited the expression of mTOR. The full-length gels of different protein expression in Western blot were shown in Supplementary Information. (**c**) Cell proliferation was inhibited after transfection with si-circ_0037251 in glioma cell lines. **P < 0.05 [All figures shown are representative. The number of technical replicates (n value) was six for each experiment. Error bars were obtained based on the standard deviation].

**Figure 2:**
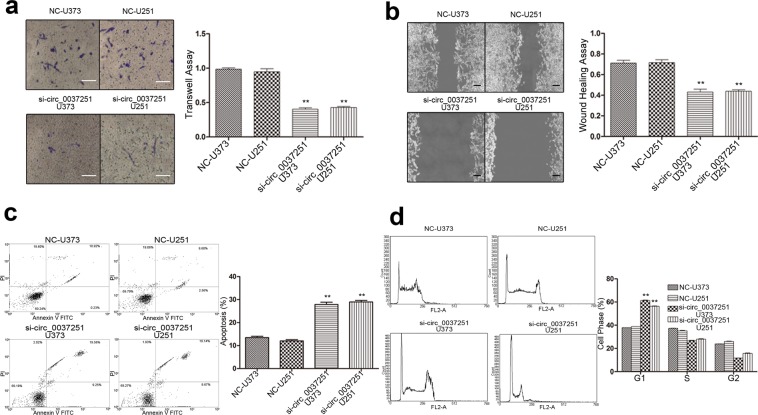
The over-expressed hsa_circ_0037251 was related to the promoted cell invasion and migration in glioma, while knockdown of hsa_circ_00037251 promoted glioma cell apoptosis, induced G1 phase arrest. (**a**) Transwell assays (magnification: ×200; scale bar: 100 μm) show that cell invasion ability was inhibited following transfection with si-circ_0037251 in glioma cell lines. (**b**) Wound healing assays (magnification: ×100; scale bar: 50 μm) show that cell migration ability was inhibited following transfection with hsa_circ_00037251 siRNAs (si-circ_0037251) in glioma cell lines. (**c**) After transfection with si-circ_0037251, cell apoptosis was promoted in glioma cell lines. (**d**) G1 phase arrest was induced after transfection with si-circ_0037251 in glioma cell lines. ** *P* < 0.05 [All figures shown are representative. The number of technical replicates (n value) was six for each experiment. Error bars were obtained based on the standard deviation].

In this study, hsa_circ_00037251 expression was significantly increased in U373 and U251 cells compared to NHA cells, which is a normal cell line (Fig. 1a). In contrast, the miR-1229-3p expression was significantly decreased in U373 and U251 cells, compared to NHA (Fig. 1a). Then, we further investigated its potential functional role by knocking down hsa_circ_00037251 in the U373 and U251 cell lines (Figs. 1c and 2). In the NC group without knockdown treatments, glioma cells exhibited significantly strong proliferation, invasion and migration abilities than other groups with hsa_circ_00037251 silencing (Fig. 1c and 2). As shown in Fig. 1c, MTT assays revealed that after transfection with si-circ_00037251, the proliferation of U373 and U251 cells was reduced compared with the NC groups (*P* < 0.01). Functionally, a flow cytometry assay revealed that glioma cell apoptosis and G1 phase arrest were promoted compared with NC group (Fig. 2) (*P* < 0.01).

Meanwhile, after transfection with si-circ_00037251, significantly increased miR-1229-3p levels were observed in glioma cell lines (Fig. 1a) (*P* < 0.01). Moreover, transfection with si-circ_00037251 also led to a significant decrease in the expression of mTOR in the expression levels of mRNA and protein (Fig. 1b) (*P* < 0.01).

### Hsa_circ_00037251 acts as a molecular sponge for miR-1229-3p, and mTOR is directly targeted by miR-1229-3p

**Figure 3:**
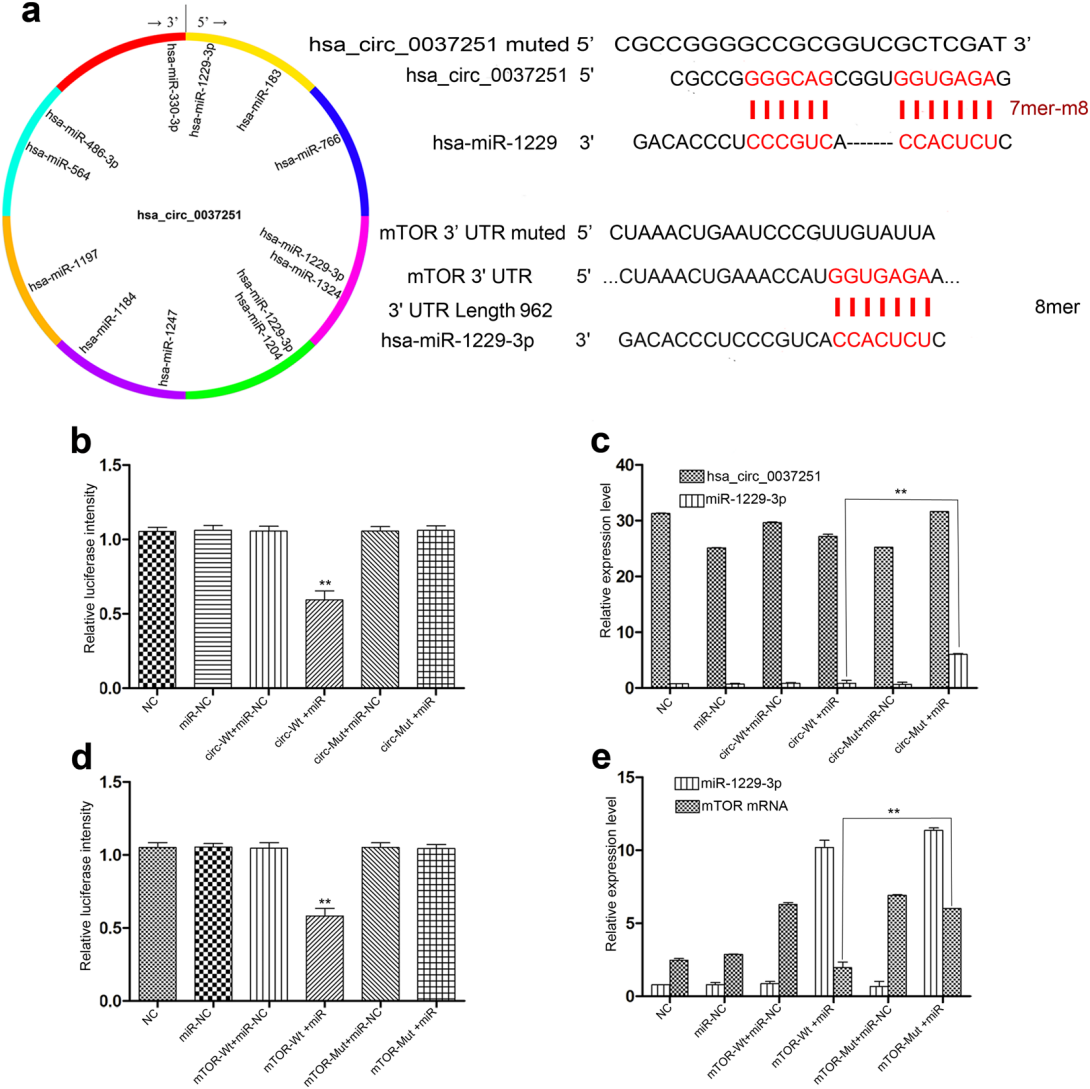
Hsa_circ_00037251 acts as a molecular sponge for miR-1229-3p, and mTOR is directly targeted by miR-1229-3p. (**a**) A schematic model showing the binding sites for hsa_circ_0037251 and miRNAs: The predicted miR-1229-3p (miR) binding site in the wild-type hsa_circ_0037251 sequence (Circ-Wt) and the mutant sequence (Circ-Mut); the predicted miR-1229-3p binding site in mTOR (mTOR-Wt) and the mutant sequence (mTOR-Mut). (**b**) Luciferase reporter assays show that the luciferase intensity was obviously reduced in the Circ-Wt + miR group. (**c**) The expression levels of hsa_circ_0037251 and miR-1229-3p in 293T cells show that the detected expression levels of miR-1229-3p in the Circ-Wt + miR group were quite different compared to the Circ-Mut + miR group. (**d**) Luciferase reporter assays show that the luciferase intensity was obviously reduced in the mTOR-Wt + miR group. (**e**) The expression levels of miR-1229-3p and mTOR in 293T cells show that the detected expression levels of mTOR mRNA in the mTOR-Wt + miR group were quite different compared to the mTOR-Mut + miR group. **P < 0.05 [All figures shown are representative. The number of technical replicates (n value) was six for each experiment. Error bars were obtained based on the standard deviation].

Luciferase reporter assays were used to determine whether hsa_circ_0037251 and the 3′-untranslated region (UTR) of mTOR share MREs for miR-1229-3p (Fig. 3a). Co-transfection of luciferase reporters containing a wild type 3′UTR sequence and miR-1229-3p mimics (miR-1229-3 pm) into 293T cells reduced the luciferase intensity by approximately 40% (Fig. 3b). We found that co-transfection of miR-1229-3 pm and the mutated luciferase reporter had no significant effect on luciferase activity (Fig. 3b). Additionally, we measured the relative expression levels of hsa_circ_0037251 and miR-1229-3p in 293T cell lines using the ^ΔΔ^Ct method (Fig. 3c).

mTOR was predicted as a direct target of miR-1229-3p (Fig. 3a). Luciferase reporter assays of 293T cells co-transfected with mTOR (wild type or mutated 3′UTR) and miR-1229-3p mimics were performed (Fig. 3d). The luciferase activity in the group with wild type 3′UTR and miR-1229-3p was significantly attenuated than that in other groups while the luciferase activity in the group with the mutated 3′UTR and miR-1229-3p was not affected (Fig. 3d). Moreover, RT-PCR was used to determine the expression of mTOR and miR-1229-3p in the 293T cell lines (Fig. 3e).

### The miR-1229-3p/mTOR axis had a key role in influencing the progression of glioma cells, and promotion of glioma cell progression can be rescued after transfection with mTOR overexpression vectors (OV)

**Figure 4:**
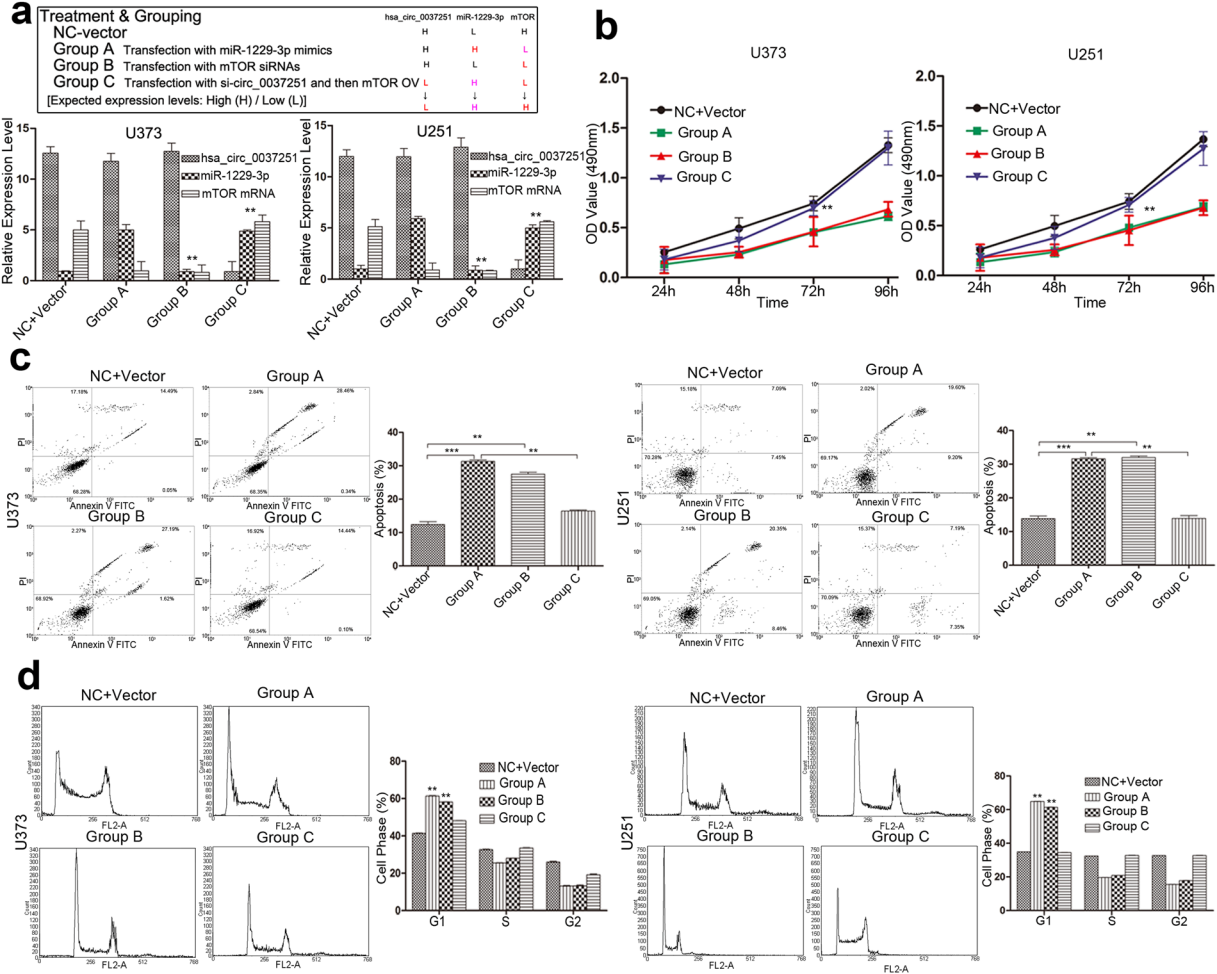
The miR-1229-3p/mTOR axis had a key role in influencing glioma cell proliferation, cell apoptosis and G1 phase arrest, and promotion of glioma cell proliferation can be rescued after transfection with mTOR overexpression vectors (OV). (**a**) The treatment and grouping methods and the expression levels in different groups were shown. Group C with rescue experiments were first transfected with hsa_circ_00037251 siRNAs (si-circ_0037251) and then transfected with mTOR OV. (**b**) Cell proliferation abilities were significantly different after different transfection assays in glioma cell lines. Results in Group A show that glioma cell proliferation was inhibited after treatments with miR-1229-3p mimics. Glioma cell proliferation can be restored following transfection of mTOR OV. (**c**) The levels of cell apoptosis were significantly different after different transfection assays in glioma cell lines. Results in Group A show that glioma cell apoptosis was promoted after treatments with miR-1229-3p mimics. Glioma cell apoptosis can be inhibited following transfection of mTOR OV. (**d**) The levels of G1 phase arrest were significantly different after different transfection assays in glioma cell lines. Treatments with miR-1229-3p mimics promoted G1 phase arrest in glioma cell lines. G1 phase arrested can be inhibited following transfection of mTOR OV. **P < 0.05, ***P < 0.01 [All figures shown are representative. The number of technical replicates (n value) was six for each experiment. Error bars were obtained based on the standard deviation].

**Figure 5:**
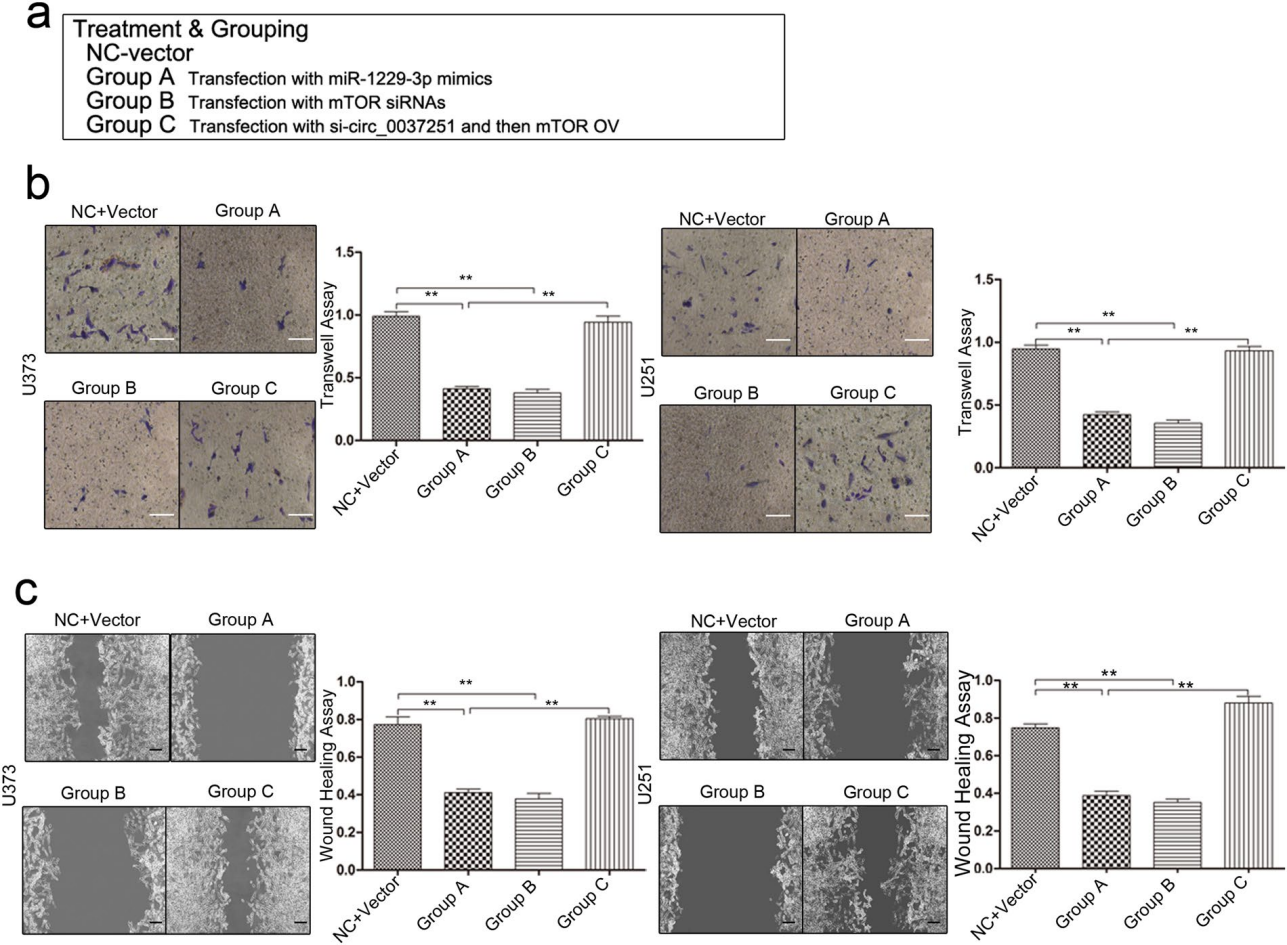
The miR-1229-3p/mTOR axis had a key role in influencing glioma cell invasion and migration abilities, and promotion of glioma cell invasion and migration can be rescued after transfection with mTOR overexpression vectors (OV). (**a**) The treatment and grouping methods and the expression levels in different groups were shown. (**b**) Cell invasion abilities were significantly different after different transfection assays (magnification: ×200; scale bar: 100 μm) in glioma cell lines. Treatments with miR-1229-3p mimics inhibited glioma cell invasion. Glioma cell invasion can be restored following transfection of mTOR OV. (**c**) Cell migration abilities were significantly different after different transfection assays in glioma cell lines. Treatments with miR-1229-3p mimics inhibited glioma cell migration (magnification: ×100; scale bar: 50 μm). Glioma cell migration can be restored following transfection of mTOR OV. **P < 0.05 [All figures shown are representative. The number of technical replicates (n value) was six for each experiment. Error bars were obtained based on the standard deviation].

Based on the interaction of hsa_circ_00037251, miR-1229-3p and mTOR, we next assessed the potential functional role of miR-1229-3p/mTOR axis by transfecting miR-1229-3p mimics and mTOR siRNAs (Fig. 4a). As shown in Fig. 4b–d, enforced expression of miR-1229-3p or reduced mTOR expression significantly promoted glioma cell apoptosis, induced G1 phase arrest and inhibited cell proliferation. In addition, the invasion and migration of cells treated with miR-1229-3pm or mTOR siRNAs were significantly inhibited compared with those in NC group (Fig. 5). Transfecting miR-1229-3p mimics and mTOR siRNAs didn’t significantly affect the expression of upstream molecules (Fig. 4a). In further rescue experiments, transfection with mTOR OV can restore the abilities of glioma cell proliferation, invasion and migration even if hsa_circ_00037251 was knocked down using siRNAs (Figs. 4 and 5).

### Hsa_circ_00037251 promoted the growth of xenografted tumours by sponging miR-1229-3p and modulating the expression of mTOR *in vivo*

**Figure 6:**
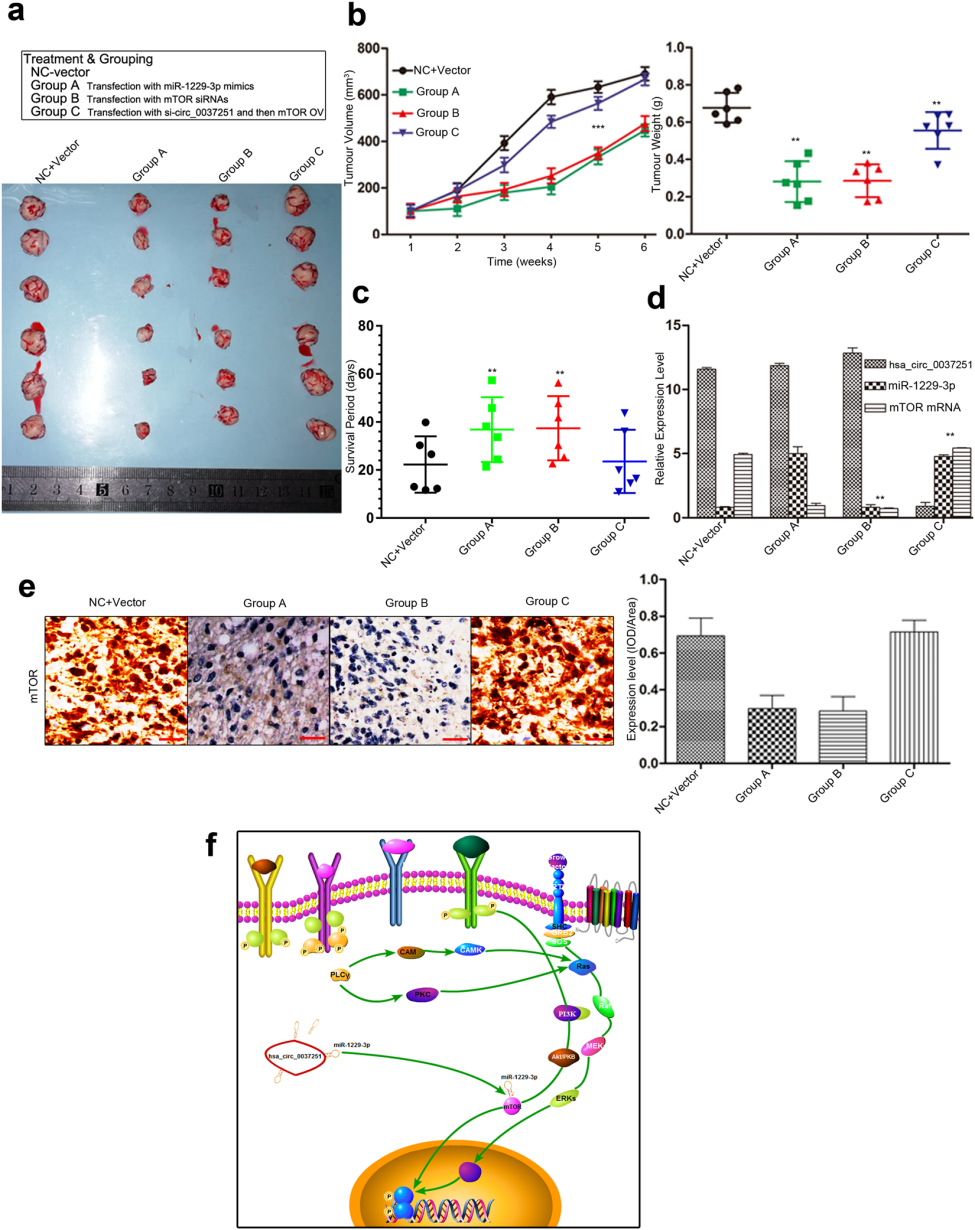
Hsa_circ_00037251 promoted the growth of xenografted tumours *in vivo*. (**a**) Representative pictures of removed xenograft tumours are presented. (**b**) Summery of tumour volumes and weights of mice are presented, respectively. The results in Group C suggested that tumor growth can be restored following transfection of mTOR OV. (**c**) The period of survival was significantly shorter in Group C than that in Group A and Group B. Of the total of 24 nude mice, only 4 nude mice (two in Group A, one in Group B and one in Group C) survived for more than 40 days. (**d**) The relative expression levels of hsa_circ_0037251, miR-1229-3p and mTOR mRNA in xenograft tumours are presented. (**e**) The expression of mTOR protein was also detected by immunohistochemistry (magnification: ×200; scale bar: 100 μm), and integrated optical density (IOD/Area) was calculated. In Group C, mTOR protein was highly expressed. (**f**) The schematic cartoon of the mechanism of hsa_circ_0037251 via miR-1229-3p in glioma cells was shown. **P < 0.05, ***P < 0.01 [All figures shown are representative. The number of technical replicates (n value) was six for each experiment. Error bars were obtained based on the standard deviation].

The *in-vivo* experiment showed that knockdown of hsa_circ_0037251or up-regulation of miR-1229-3p resulted in a smaller tumour volume and decreased the less tumour weight (Fig. 6a,b). The survival analysis demonstrated that hsa_circ_0037251 inhibition or miR-1229-3p reintroduction led to longer survival (Fig. 6c). In addition, the rescue experiments promoted glioma tumour growth (Fig. 6a–c) and shortened the survival period in nude mice (Fig. 6c). The expression levels of hsa_circ_0037251 and miR-1229-3p were also detected in the removed tumours as well as that of mTOR (Fig. 6d,e).

## Discussion

Glioma, with a high recurrence rate, is one of the most frequently occurring subtypes of brain tumours^1–3^. To reveal the mechanism underlying the glioma progression and discover new reliable therapeutic targets, an increasing number of basic studies have focused on circRNAs^22,23^.

Functional and molecular experiments revealed that hsa_circ_0037251 may have a positive effect on glioma cell progression. In this study, we observed dramatic overexpression of hsa_circ_0037251 in glioma cell lines. The NC groups with high expression levels of hsa_circ_0037251 had stronger capabilities for proliferation, invasion and migration than other groups. Knockdown of hsa_circ_0037251 induced cell apoptosis and G1 phase arrest, suggesting its tumour-promoting effect. Similar tumour promotion has also been observed in other studies^22,23^. Recent studies have also shown that other circRNAs serve as regulators in glioma tumourigenesis^22,23^, and this is the first report on regulatory function of hsa_circ_0037251 in glioma. Meanwhile, *in vivo* experiments also supported the tumour-promoting function of hsa_circ_0037251. These results indicated that the progression of glioma was effected by multiple circRNAs, and hsa_circ_0037251 may be an important promotor in glioma.

Furthermore, hsa_circ_00037251 acts as a molecular sponge for miR-1229-3p. Unlike the results of expression analysis in other tumours^24^, miR-1229-3p was obviously down-regulated in glioma tissues and cell lines. This expression is inconsistent with the results of the study by Butkyte *et al*.^24^. In their research, the expressions of miR-1229-3p were generally up-regulated in other tumours^24^. To further clarify the specific role of miR-1229-3p, microarray analysis on miRNAs and genes were performed. Gradually, the sponging effect of hsa_circ_0037251 on miR-1229-3p was confirmed through luciferase reporter assays. There was a negative correlation between the expression levels of hsa_circ_0037251 and miR-1229-3p, and the expression of miR-1229-3p was promoted by knockdown of hsa_circ_00037251. Similarly, in a recent study, a circRNA named circRNA_100290, was reported to have sponging effects on other miRNAs in glioma^25^. Another circRNA called circ-ITCH was also reported to serve as a sponge for miR-17/miR-224 in glioma^26^. Thus, the sponging effect was common in gliomas and may be one of the important mechanisms that promote the progression of glioma.

Moreover, hsa_circ_0037251 may exert its regulatory functions through miR-1229-3p/mTOR axis. In this regulatory axis, the proliferation, invasion and migration capabilities of glioma cells were significantly altered by regulating the expressions of miR-1229-3p and mTOR while transfection with miR-1229-3p mimics or mTOR siRNAs significantly promoted glioma cell apoptosis and G1 phase arrest. These results indicated that miR-1229-3p/mTOR axis has an indispensable inhibitory effect on the circRNA-miRNA regulation of glioma progression. The similar tumour-promoting circRNA-miRNA-gene regulatory axis can also be detected in other tumours^25^. For insistence, Chen *et al*. demonstrated that circRNA_100290 may function as an endogenous tumour-promoting RNA to regulate CDK6 expression through sponging up miR-29b family members in oral cancer^25^. However, Yang *et al*. discovered that another sort of circRNA-miRNA-gene axis showed inhibitory effect in tumour progression^26^. They stated that circ-ITCH acts as a tumour suppressor by a circ-ITCH/miR-17, miR-224/p 21, PTEN axis in bladder cancer^26^. Therefore, from the above aspects, it may be the specific regulatory axis that ultimately determined the role of hsa_circ_0037251.

In addition, the presented results also revealed that mTOR is the downstream target of the circRNA-miRNA network, and the tumour-promoting function on glioma can be rescued after transfection with mTOR OV. As expected in our hypothesis concerning the regulatory network, we found that inhibition of hsa_circ_0037251 increased the expression of miR-1229-3p but decreased the expression of mTOR. And mTOR was identified as a target of miR-1229-3p such that up-regulation of miR-1229-3p reduced expression of mTOR. Moreover, some other studies have reported that up-regulation of mTOR signaling might promote tumourigenesis^27,28^ and inhibition of mTOR complexes blocks the cell cycle and induces apoptosis^28–30^. Although glioma cells with under-expressed hsa_circ_0037251 or over-expressed miR-1229-3p exhibited the down-regulated abilities of progression, capabilities for proliferation, invasion and migration were significantly restored by transfection with mTOR OV. These indicated that the inhibition of si-circ_0037251 was reversed by mTOR OV in the rescue experiment. It can be seen that, as a downstream target, mTOR was one of the direct factors affecting the progression of glioma, no matter what expression levels of hsa_circ_0037251 and miR-1229-3p exhibited.

However, more effective, accurate and specific methods of RNA interference remain to be exploited. And further specific studies are needed to determine whether hsa_circ_0037251 plays a role in some other pathological processes. Moreover, the more rigorous experimental conditions are necessary to investigate the distributions of mTOR in the cytoplasm and nucleus. Nevertheless, our study was the first to investigate the role and mechanism of circular METRN RNA including hsa_circ_0037251 in glioma, and revealing the role of circRNAs will be critical for understanding glioma pathogenesis and offering novel insight into the identification of new biomarkers or new potential therapeutic targets of glioma.

In summary, the results indicated that hsa_circ_0037251 may exert its regulatory functions in glioma progression through sponging miR-1229-3p and finally modulated the expression of mTOR. The identified hsa_circ_0037251/miR-1229-3p/mTOR axis may provide a potential biomarker and therapeutic target for glioma.

## Methods

### Cell culture

Human U373, U251 glioma cell lines and human embryonic kidney (HEK) 293T cells were purchased from the Cell Resource Center of the Shanghai Institute of Biological Sciences. We also purchased primary normal human astrocytes (NHA) from Sciencell Research Laboratories (Carlsbad, CA, USA). Glioma cells were cultured in DMEM medium (Gibco, USA) containing 10% fetal calf serum at 37 °C and placed in a humidified atmosphere containing 5% CO_2_. The cells of the same batch were stably subcultured for 5 generations. Cell lines retained before and after stable subculture were further analyzed within 6 months.

### Wound healing assay

The cells were seeded in 6-well plates (4 × 10^5^ cells/well). When the cell confluence reached 80% after 24 hours, the cells were treated with mitomycin C (1 µg/ml; Sigma-Aldrich; Merck KGaA) at 37 °C for 1 h. Then, we created wounds on the surface of the cell monolayer by using a 200-µl pipette tip. Cells were cultured in a cell incubator and an inverted microscope (Motic Instruments, Richmond, BC, Canada) was used for image acquisition with magnification of ×100 at 24 h. The relative migration ratio was also calculated [Relative migration ratio = (incipient gap between two edges- migrated gap between two edges)/incipient gap between the two edges].

### Transwell assay

Transwell inserts and Matrigel were purchased from Corning Incorporated (Corning, NY, USA) and BD Biosciences (San Jose, CA, USA), respectively. We added a total of 4 × 10^3^ cells in 200 µl cell medium into Transwell inserts that were precoated with Matrigel, and then added 800 µl medium supplemented with 30% FBS into the lower chambers. Thereafter, the cells were transfected and cultured in a cell incubator and allowed to invade for 24 hours. We removed cells on top of the membranes after rinsing. Cells (invaded through the membranes) were treated with 4% paraformaldehyde at room temperature for 20 min and stained by 0.5% crystal violet for 5 min. An inverted microscope (Motic Instruments, Richmond, BC, Canada) was applied in capturing images with a magnification of ×200.

### Luciferase reporter assay

A total of 3 × 10^4^ 293T cells were seeded in 24-well plates in triplicate. Luciferase reporter assays were conducted using a Dual-Luciferase reporter assay system (Promega, Madison, WI) 48 hours after co-transfection with corresponding plasmids and miRNA mimics or inhibitors according to the manufacturer’s instructions. Relative luciferase activity was normalized according to the internal control of Renilla luciferase.

### Quantitative real-time PCR (RT-PCR)

MiRNA concentrations were determined using an ABI PRISM7900 system (Applied Biosystems, Carlsbad, CA, USA), and quantification of circRNA and mRNA was performed using an ABI PRISM7500 system. Before calculation using the ΔΔCt method, the levels of small nuclear U6 were used to normalize the miRNA expression levels, and the levels of GAPDH were used to normalize the relative expression levels of circRNA and mRNA. The primers used for quantitative real-time PCR were as follows: hsa_circ_0037251, 5′-CTGCTTTGGAGGTGATGGGAC-3′ (forward) and 5′-GGGAGTCGGGGCTCTCAC-3′ (reverse); miR-1229-3p, 5′-CCACTGCCCTCCCA-3′ (forward) and 5′-GGTCCAGTTTTTTTTTTTTTTTCTGT-3′ (reverse); mTOR, 5′-CTGGGACTCAAATGTGTGCAGTTC-3′ (forward) and 5′-GAACAATAGGGTGAATGATCCGGG-3′ (reverse). The expressions of hsa_circ_0037251 and miR-1229-3p were detected in the U373, U251, NHA cell lines before the beginning of subculture (line0) and after the stable subcultures (line1) in our laboratory.

### MTT assay

inCells were collected by centrifugation after incubation with 5.0 mg/mL 3-(4, 5-dimethylthizol-2-yl) 22, 5- diphenyltetrazolium bromide (MTT) at the indicated time point post-transfection. Dimethyl sulfoxide (200 μL) was added into the sediments, and then the absorbance was measured by spectrophotometry at 490 nm.

### Cell cycle and apoptosis assays

The transfeted cells were stained with propidium iodide for the cycle test plus a DNA reagent kit (BD Biosciences, USA) and then measured by flow cytometry (Mindray, China). The cells ratios in the G1, S, and G2 phases were counted and compared. To identify cell apoptosis, cells were also stained using an annexin V apoptosis kit (eBiosciences, USA) and analyzed using flow cytometry.

### Western-blot analysis

In order to detect the protein expressions of mTOR, phospholipase C gamma 1 (PLCG1) and SHC adaptor protein 1 (SHC1), total proteins were extracted using lysis buffer, separated on 12% SDS-PAGE gels and blotted on cellulose membranes. The membranes were immunoblotted with a secondary antibody at room temperature for 1 hour after hybridization with a monoclonal antibody at 4 °C overnight. Finally, enhanced chemiluminescence (ECL kit, Santa Cruz Biotechnology) was used for visualization and a Quantity One system (Bio-Rad, Hercules, CA, USA) was applied for analysis. Primary antibodies included anti-mTOR (dilution 1:200, Santa Cruz Biotechnology), anti-PLCG1 (dilution 1:1000, ThermoFisher Scientific), anti-SHC1 (dilution 1:2000, Abnova) and anti-GAPDH (dilution 1:1000, Santa Cruz Biotechnology).

### RNA interference and transfection assay

Small interfering RNAs (siRNAs) targeting the back-splice junction of hsa_circ_0037251 (si-circ_0037251) were designed and synthesized by RiboBio (Guangzhou, China). For si-circRNA, the functional sequence of the sense strand was 5′-AUGCACCAGCGACUUCGUAAU-3′ and the antisense strand sequence was 5′-ATTACGAAGTCGCTGGTGCAT-3′. Based on the manufacturer’s protocol, cells were transfected using Lipofectamine 2000 (Invitrogen, Carlsbad, CA, USA).

### Xenografts in mice

After the glioma cells were stably transfected, approximately 1 × 10^7^ cells were injected into BALB/C nude mice (4–6 weeks old, 18–22 g, six female mice per group). Tumour growth was detected every week by monitoring the width (W) and length (L) with calipers, and the tumour volume (V) was calculated using the formula V = (W^2^ × L)/2. Tumours were measured regularly, and the mice might be euthanized as planned 10 weeks after injection. All animals’ experimental protocols were approved by the Ethics Committee of Zhengzhou University. All methods were carried out in accordance with the relevant guidelines and regulations. Every effort was made to minimize the number of animals used and their suffering.

### Immunohistochemistry assays

Before blocking with 10% normal goat serum (MXB, Fuzhou, China) for 30 min and incubating overnight at 4 °C with rabbit polyclonal antibody against mTOR (1:150, SAB, Chicago, IL), the slides (4 μm thick) of xenograft samples were dewaxed, rehydrated, and incubated in 0.3% H_2_O_2_ for 10 min to inhibit endogenous peroxidase activity. Slides were washed with PBS three times and then incubated with biotinylated rabbit anti-rabbit IgG for 1 hour at room temperature. For each sample, integrated optical density (IOD/Area) was also calculated (Image-Pro Plus 6.0, Media Cybernetics, Rockville, MD, USA) and employed as mTOR expression level.

### Statistical analysis

Experimental data are represented as the mean ± standard deviation (SD). Error bars were obtained based on the standard deviation. Student’s two-tailed unpaired t test was used to determine the statistical significance of the *in vitro* experiments. All statistical tests were two-sided, and a *P* value < 0.05 was considered statistically significant.

## Supplementary information

SupplementaryFigureS1.
