# Supplementary material for: Circular METRN RNA hsa_circ_0037251 Promotes Glioma Progression by Sponging miR-1229-3p and Regulating mTOR Expression
Source: Sci Rep. 2019 Dec 24;9:19791. doi: 10.1038/s41598-019-56417-8 (PMC6930248; doi:10.1038/s41598-019-56417-8)

Supplementary Information

Circular METRN RNA hsa_circ_0037251 Promotes Glioma Progression by Sponging miR-1229-3p and Regulating mTOR Expression

Qinchen Cao[1*], Yonggang Shi[1], Xinxin Wang[2], Jing Yang[1], Yin Mi[1], Guan Zhai[3], Mingzhi Zhang[4]

[1]Department of Radiation Therapy, The First Affiliated Hospital of Zhengzhou University, Zhengzhou 450052, People's Republic of China

[2]Department of Neurology, The First Affiliated Hospital of Zhengzhou University, Zhengzhou 450052, People's Republic of China

3 Department of Neurosurgery, The First Affiliated Hospital of Zhengzhou University, Zhengzhou 450052, People's Republic of China

[4]Department of Oncology, The First Affiliated Hospital of Zhengzhou University, Zhengzhou 450052, People's Republic of China

[*]Correspondence: Dr. Qinchen Cao, Department of Radiation Therapy, The First Affiliated Hospital of Zhengzhou University, Zhengzhou 450052, People's Republic of China.
E-mail: fcccaoqs@zzu.edu.cn   Fax: +86371-67966842/+86371-67966841

Supplementary Figure S1. Full-length blots of Figure 1.

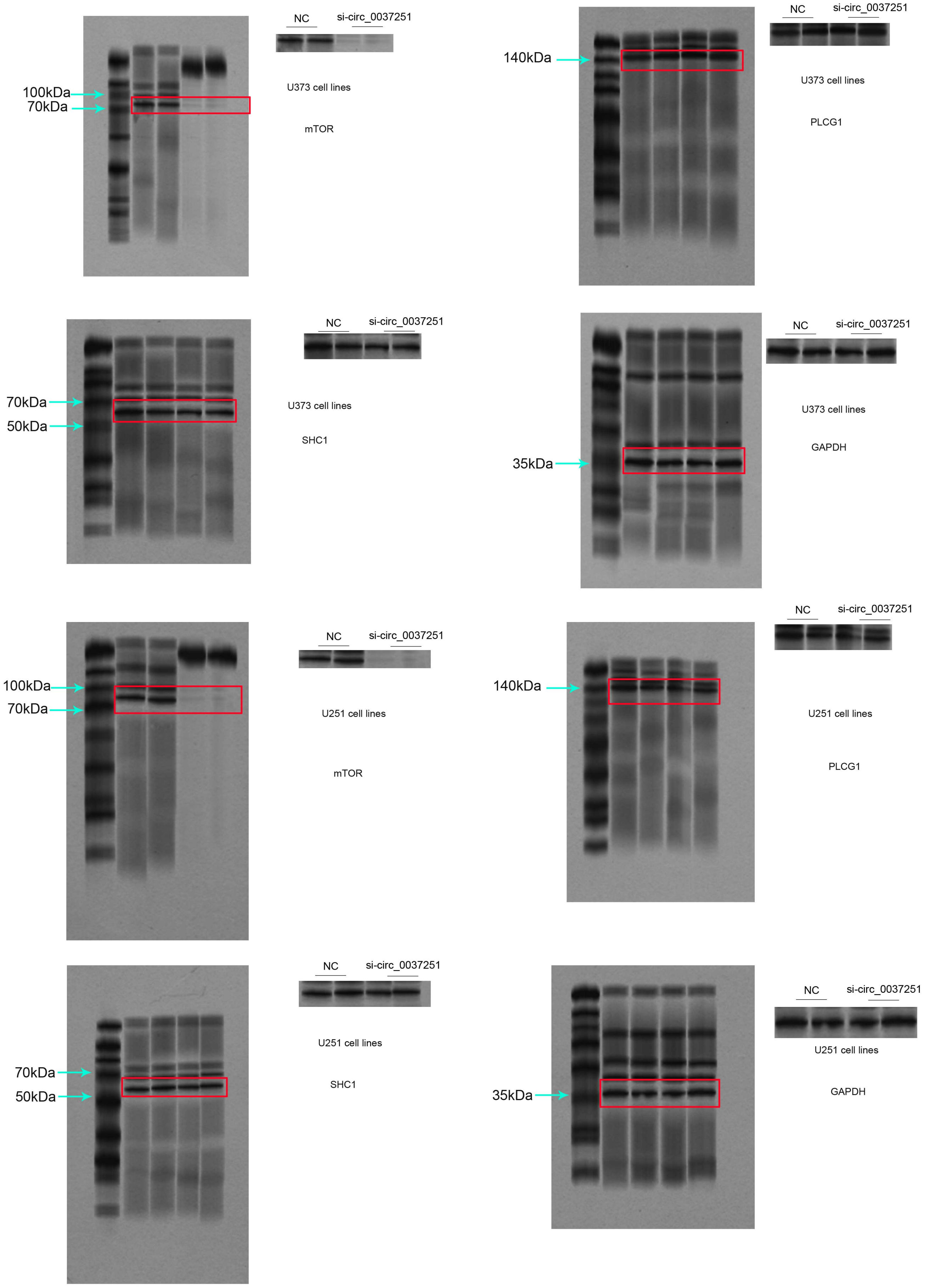

Supplement: Supplementary file 1 — SupplementaryFigureS1. [file 41598_2019_56417_MOESM1_ESM.pdf]
